# Skin Blood Flowmotion in Various Disease States: A Scoping Review

**DOI:** 10.7759/cureus.101379

**Published:** 2026-01-12

**Authors:** Melanie Rodriguez, Lily Tehrani, Harvey N Mayrovitz

**Affiliations:** 1 Medicine, Dr. Kiran C. Patel College of Osteopathic Medicine, Nova Southeastern University, Fort Lauderdale, USA; 2 Medical Education, Dr. Kiran C. Patel College of Allopathic Medicine, Nova Southeastern University, Davie, USA

**Keywords:** blood flow, blood velocity, diabetes, flow motion, hypertension, inflammatory conditions, laser-doppler flowmetry, microcirculation, vasomotion

## Abstract

Skin blood flowmotion (FM) describes the spontaneous rhythmic changes in microvascular blood flow that occur as blood vessels dilate and constrict over time. These oscillations could occur secondary to several physiological influences, and their frequencies are often expressed as distinct frequency bands associated with cardiac activity, respiration, myogenic tone, neurogenic input, and endothelial factors. Laser Doppler flowmetry (LDF) is commonly used to measure these patterns and to analyze their frequency components. This scoping review examines peer-reviewed English-language studies on FM to better understand its physiological basis and potential relevance across various disease states. A systematic search of Embase, OVID, and Web of Science identified 445 unique articles that used LDF to evaluate FM in relation to a disease state. After screening titles and abstracts of English-language articles involving human subjects, relevant full texts were independently reviewed by two investigators, with a third reviewer resolving disagreements. Studies that focused only on healthy subjects or did not measure FM were excluded. The 41 studies that were included in the review identified several frequency bands in LDF recordings that reflect distinct regulatory mechanisms across disease states. Findings also show that factors such as sympathetic nerve activity, endothelial function, the anatomical site of measurement, and underlying disease can alter these FM oscillatory patterns and may be useful for early disease recognition. The literature also indicates that FM reflects multiple layers of microvascular regulation and may provide useful insights into early or subtle microcirculatory changes. Extended research is warranted that uses more standardized measurement approaches to further clarify clinical significance.

## Introduction and background

Normal features of flowmotion (FM)

**Figure 1:**
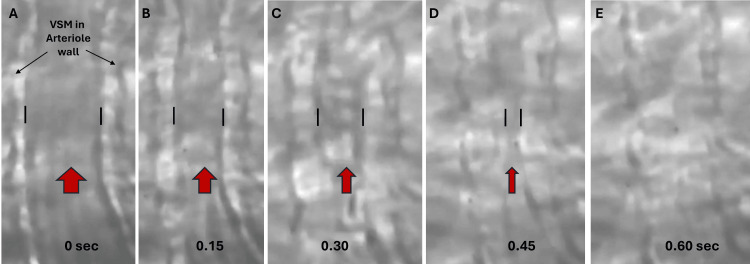
Illustrating flowmotion in an arteriole The vascular smooth muscle (VSM) lining the walls of the arteriole is brighter than the blood flowing through its lumen. The pair of vertical black lines defines the width of the lumen, which is seen to decrease from panel A through panel D. Panel E shows that the contraction of the VSM has wholly obliterated the lumen. The overall contraction process from A through E takes 0.60 seconds. The blood flow, shown with the red arrows, is vigorous in A, diminished from B through C, and ultimately stopped in E. This process is transient, and the lumen is again vasodilated, progressing from E through A. These blood flow dynamics are flowmotion. The figure is provided as a courtesy of Dr. HN Mayrovitz. The full video is available at https://www.youtube.com/user/hnm1313.

The term blood FM refers to spontaneous changes in blood flow within the microcirculation. These changes occur due to simultaneous changes in the lumen dimension of blood vessels within the microvascular network. This process is illustrated in Figure 1, with a sequence of consecutive microscopic images captured from an educational video illustrating spontaneous vasomotion in a small arteriole [1]. 

**Figure 2:**
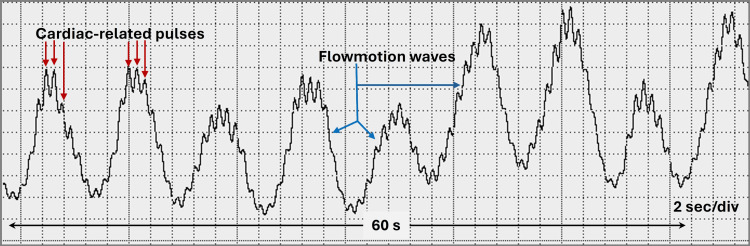
Illustrating flowmotion waves obtained using Laser Doppler flowmetry. The large excursions are flowmotion waves, which here occur at about 8 waves/minute. The superimposed minor excursions are due to cardiac pulses, which occur at a rate of about 60 per minute. The figure is provided as a courtesy of Dr. HN Mayrovitz.

The impact that these rhythmical, often spontaneous blood flow changes have on various microvascular functions has been studied using mathematical models [2], animal models [3], and in humans [4]. When studied in humans, it is often measured in the skin using laser Doppler flowmetry (LDF), which provides recordings of the time-varying flux of red blood cells (RBCs) within the microvascular network [5-9]. Figure 2 illustrates a segment of such a recording that is typical of those seen in the normal skin of the foot.

Spectral characterization of flowmotion

Much research has been done to characterize the features of such FM and to investigate the factors responsible for their occurrence and variation. One characterization method used is spectral analysis of the LDF signal to obtain characteristic FM frequencies using either wavelet analysis [9,10] or Fourier methods [11,12]. The outcomes of these analyses demonstrated that the LDF signal had spectral power across several frequency bands. It has been reported that wavelet transforms allow for better frequency discrimination at lower frequencies [13]. When applied to LDF signals, five frequency bands were identified, with spectral power peaks at about 1 Hz, 0.3 Hz, 0.1 Hz, 0.04 Hz, and 0.01 Hz, as shown in Table 1. The 1 Hz peak was due to cardiac pulsations; the 0.3 Hz peak was due to respiratory activity; the 0.1 Hz peak was interpreted as myogenic activity of vascular smooth muscle (VSM) in blood pressure regulation; and the 0.04 Hz peak was interpreted as neurogenic activity. The 0.01 peak was hypothesized to be associated with metabolic regulation of the vasculature [13]. In healthy people, spectral amplitude was reported to increase only in the 0.01 Hz band when the skin was exposed to endothelial-dependent vs. independent vasodilators [14]. This suggested that this band was associated with endothelial metabolic activity. These concepts are summarized in Table 1 based on literature reports [15].

**Table 1:** Characteristic frequency bands in laser Doppler flowmetry (LDF) signal analysis and their physiological origins [15].

Frequency (Hz)	Cause of Flowmotion	Description
0.6-2.0	Cardiac	Heartbeats
0.145-0.60	Respiratory	Breathing
0.052-0.145	Myogenic	Vessel muscle tone (smooth muscle regulating blood pressure)
0.021-0.052	Neurogenic	Nerve signals to vessels
0.005-0.021	Endothelial	Vessels responding to metabolic demands, nitric oxide release

Factors affecting flowmotion patterns

Direct measurements of changes in the diameters of small arterioles have been shown to correlate with FM frequencies of about 0.1 Hz in an experimental animal model, indicating a connection between vasomotion and FM. Several physiological factors have been identified that influence both the amplitude and spectral content of skin blood flow when assessed using LDF methods [16-18]. By comparing LDF patterns on intact skin with those on nearby skin lacking sympathetic nerve activity (SNA), it was demonstrated that SNA was responsible for the spectral content between 0.02 Hz and 0.05 Hz. This observation was likely due to SNA's effects on the VSM of arterioles. In contrast, part of the oscillatory flow pattern at 0.01 Hz has been attributed to the release of nitric oxide (NO) from endothelial cells. This lower-frequency dynamic may be due to the longer time constant associated with NO's action on VSM. Changes in the spectral content of several frequency bands have also been reported to be significantly affected by general anesthesia [19]. 

A somewhat different approach to determining the physiological basis for the various spectral variables has been undertaken using wavelet analysis combined with correlational calculations [20]. LDF and skin temperature measurements on the hand dorsum of 34 healthy volunteers suggested that opening and closing of skin arteriovenous shunts in response to temperature changes played a significant role, accounting for 69% of the relative contribution to LDF oscillations. However, a more substantial role may be present since a strong relationship has been reported between the spectral content of skin blood flow and oxygen saturation [11]. It has also been reported that the anatomical location of LDF assessment may yield different FM characteristics when measured on the forehead or face [21]. 

Study goals

Despite considerable uncertainty about the normal physiological origin and effects of FM associated with vasomotion, various features have been investigated in conditions including hypertension (HTN) [22], diabetes [23], and vascular disease [24], to name a few. The present scoping review aims to evaluate and synthesize existing literature on skin blood FM characteristics, focusing on their physiological significance and potential clinical utility in diagnosing and treating disease.

## Review

Methods

Search Strategy 

The information provided below was gathered from three databases: Embase, OVID, and Web of Science. A step-by-step screening process was carried out, in which two reviewers independently evaluated article titles and abstracts for relevance. Full-text reviews were performed for studies that met the inclusion criteria. If disagreements arose, a third reviewer was consulted to resolve the issue. The search utilized Boolean operators ‘AND’ and ‘OR’ to combine selected search terms: (“skin flowmotion” OR “skin vasomotion” OR “laser Doppler” OR “cutaneous blood flow” OR “skin microcirculation”) AND (“vasomotion” OR “flowmotion” OR “blood flow oscillations” OR “microvascular regulation”) AND (“healthy” OR “control subjects” OR “disease” OR “diabetes” OR “hypertension” OR “vascular dysfunction” OR “endothelial function” OR “systemic disease” OR “microcirculatory impairment”). No date limitations were applied during the initial search to ensure a thorough review of all relevant literature. Access to full texts of restricted articles was obtained through the Nova Southeastern University (NSU) library database.

*Selection Criteria* 

**Figure 3:**
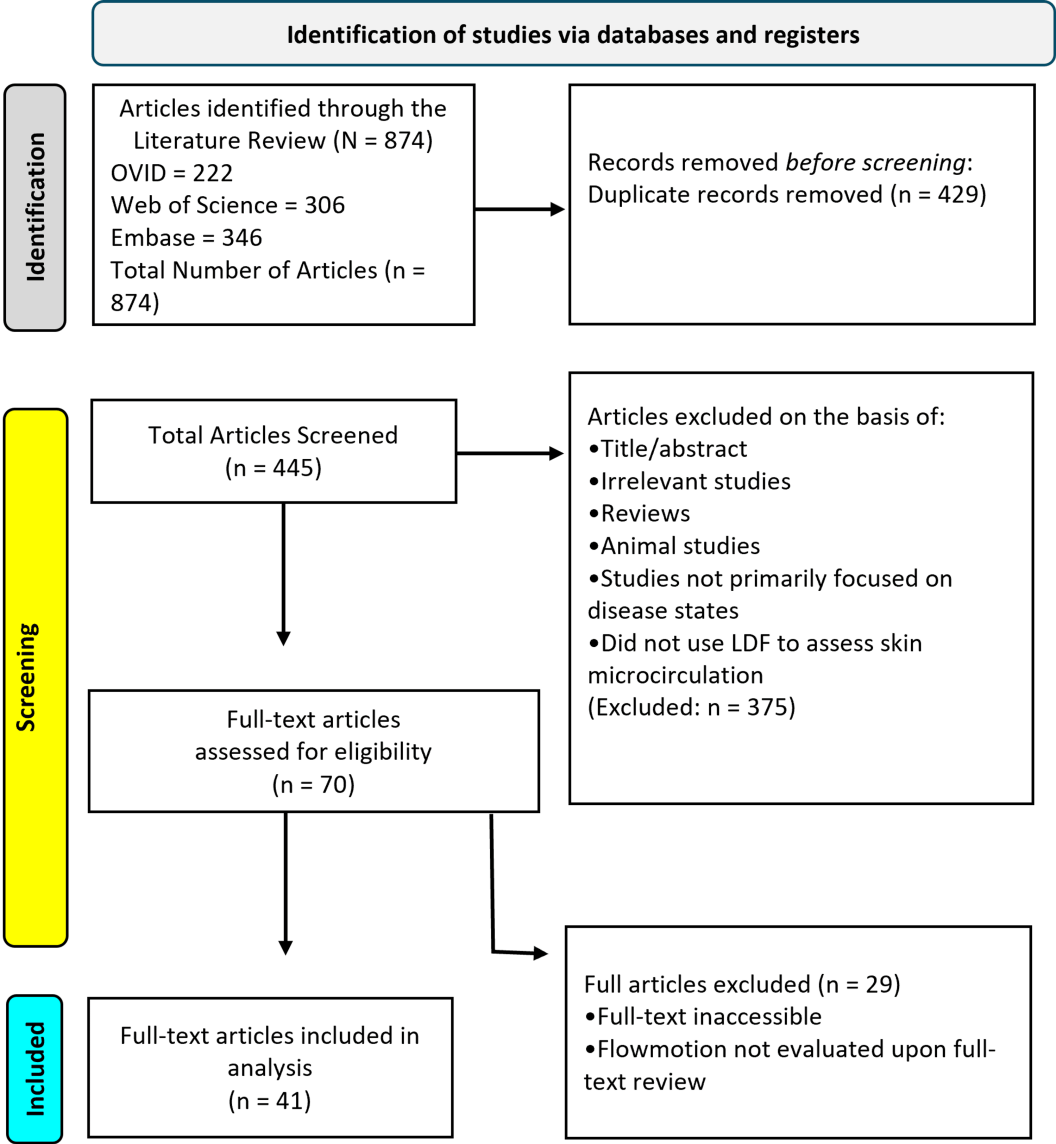
Preferred Reporting Items for Scoping reviews and Meta-Analyses (PRISMA) diagram indicating the search procedure PRISMA diagram indicating the search procedure [25].

The Preferred Reporting Items for Scoping Reviews and Meta-Analyses (PRISMA) guidelines were followed [25], and a PRISMA flow diagram (Figure 3) illustrates the screening and selection process. After removing duplicates, titles and abstracts were reviewed for relevance. Studies were eligible if they met the following criteria: published in English, involved human participants, used LDF to assess skin microcirculation, and specifically analyzed FM, defined as rhythmic oscillations in blood flow, in the context of a disease state. Studies were excluded if they focused solely on healthy subjects or measured only blood flow without assessing FM.

Results

As depicted in the PRISMA diagram, our initial database search yielded 874 articles: 222 from OVID, 306 from Web of Science, and 346 from Embase. After removing 429 duplicate entries, 445 articles remained for title and abstract screening. From there, 375 articles were excluded based on relevance and inclusion criteria. The full text of the remaining 70 studies was reviewed, and an additional 27 were excluded for reasons such as a lack of FM analysis, an ineligible study design, or the use of non-LDF methodology. In total, 41 studies met all inclusion criteria and were included in the final review.

The following part of the results describes alterations in FM across various disease states. 

Hypertension

LDF, measured on the forearm, was used to analyze FM changes in 59 supine patients with newly diagnosed systemic arterial HTN and in 30 normotensive controls [26]. HTN was defined as an average daily blood pressure of 130/80 mmHg or higher. Using 24-hour ambulatory monitoring, the hypertensive group had a median daytime blood pressure of 136/90 mmHg, while the control group had a median of 121/78 mmHg. Average skin blood perfusion was similar between groups. Still, wavelet analysis revealed a significantly reduced FM amplitude in the hypertensive group within the neurogenic, myogenic, and cardiac frequency bands (p < 0.05 for all). At the same time, no difference was observed in the endothelial and respiratory bands. Perfusion efficiency, defined as the spectral amplitude associated with each physiological mechanism relative to mean perfusion, was also significantly reduced in the hypertensive group for endothelial, neurogenic, myogenic, and cardiac bands. These findings demonstrate that HTN is linked to reductions in FM parameters across most spectral bands [26]. Additional evidence for a hypertensive connection was found in a study of 18 untreated hypertensives with an average blood pressure of 139/92 mmHg, compared with 18 age-matched normotensive controls [27]. At rest, forearm skin blood flow (baseline flux) was not significantly different between groups. However, when the skin was heated to 42°C and blood flow reached a stable plateau (reflecting NO-dependent vasodilation), hypertensive individuals showed a blunted response (139 ± 13 vs. 174 ± 18 AU, p = 0.050). Spectral analysis revealed that the hypertensive group also had lower total power spectral density (PSD) during this heating response (p = 0.03), particularly in the neurogenic frequency band (p < 0.01), with a trend toward lower endothelial activity (p = 0.07). When expressed as relative contributions, the hypertensive group had a smaller neurogenic component (p < 0.001), a greater myogenic component (p = 0.04), and an unchanged endothelial component. Following NOS inhibition with L-NAME, hypertensive individuals showed further reductions in endothelial (p < 0.01), neurogenic (p = 0.04), and total PSD (p < 0.001) compared to controls. Despite these impairments, vasodilation induced by sodium nitroprusside (SNP) was preserved in both groups, indicating intact smooth muscle function. These findings suggest that HTN is characterized by impaired neurogenic and NO-dependent FM regulation, even though smooth muscle responsiveness remains intact [27]. Additional local heating experiments studied FM during eight minutes of local heating of the forearm to 44°C in three groups: 31 patients with newly diagnosed HTN, 22 normotensives with a family history of hypertension (FHTN), and 17 normotensives without a family history of HTN (NORM) [28]. HTN was confirmed over three separate visits using ABPM, with an average daytime BP of 136/90 mmHg in the HTN group and 121/78 mmHg in normotensive controls. Although resting and peak LDF during heating were similar across all groups, cutaneous vascular conductance (CVC) at maximal heat flow (calculated as the highest LDF value during heating divided by the MAP) was significantly reduced in the HTN group compared with NORM (1.30 ± 0.53 vs. 1.87 ± 0.70 AU/mmHg, p = 0.01). Spectral analysis during the heating phase showed that FHTN subjects had significantly lower total spectral power, as well as reduced myogenic (p = 0.03), respiratory (p = 0.006), and cardiac (p = 0.02) components compared to NORM, while HTN subjects did not differ from NORM in these bands. Endothelial and neurogenic components remained unchanged across groups. These findings suggest that established HTN is linked with impaired vasodilatory efficiency during maximum heating, while individuals with a family history already show reductions in certain oscillatory components, indicating early microvascular dysfunction before overt HTN develops. 

Another study examined FM in 46 newly diagnosed, untreated hypertensive patients (HTNU) and compared them to 20 normotensive controls (NORM) using LDF recordings from the forearm at rest for 30 minutes [29]. The HTNU group showed significantly higher absolute spectral amplitudes across most frequency bands compared to controls, but the relative contribution of the myogenic band was significantly reduced (p < 0.01). After eight weeks of antihypertensive treatment in a subset of 22 patients, absolute amplitudes normalized across all bands except for the respiratory component, which remained elevated (p < 0.01). However, reduced relative myogenic activity persisted despite treatment. These findings suggest that early antihypertensive treatment may help reverse skin microvascular abnormalities, but impaired myogenic regulation may remain. A larger study compared 63 newly diagnosed hypertensive patients with 30 normotensive controls. LDF was recorded for six minutes on the forearm and analyzed using wavelet analysis, which demonstrated reduced spectral amplitudes in the endothelial and neurogenic bands (p < 0.002) [30].

A final study investigated whether coordination between cardiac, respiratory, and myogenic LDF oscillations is preserved in individuals with treated arterial HTN. The study included 73 participants divided into three groups: 29 healthy young adults (YOUNG), 22 healthy older adults (OLDN), and 22 older adults with pharmacologically treated HTN (OLDH) [31]. Simultaneous 30-minute recordings of LDF (right wrist), electrocardiogram (ECG), and respiratory effort were collected at rest. LDF spectral power in the cardiac band was significantly higher in OLDN compared with YOUNG and OLDH (p < 0.001). Respiratory and myogenic power were lower in YOUNG compared with the two older groups (p < 0.05). Coherence, defined as the synchrony between ~0.1 Hz myogenic oscillations in skin blood flow and heart rate variability oscillations, declined with age and was nearly absent in the OLDH group, suggesting impaired coordination of vascular and cardiac rhythms despite antihypertensive therapy [31]. 

Overall, the results from these HTN-related studies show that HTN is associated with decreased spectral power and perfusion efficiency in the neurogenic and myogenic frequency bands, changes in cardiac FM dynamics, and impaired coordination among microvascular oscillators, even in early or treated stages of the condition. 

Raynaud’s Phenomenon 

Raynaud’s phenomenon is associated with digital artery vasoconstriction triggered by cold temperatures or emotional stress [32]. LDF on the palmar surface of both index fingers in 61 patients with Raynaud’s phenomenon revealed variable FM patterns when the skin was heated to 40 °C and cooled to 5°C [33]. Some patients had a dominant low-frequency component of 0.038 Hz ± 0.015 Hz, which was significantly (p < 0.05) lower than the 0.054 Hz ± 0.018 Hz found in a healthy control group.

Peripheral Arterial Disease (PAD)

PAD is a condition characterized by reduced blood flow to the limbs. It is most often caused by atherosclerosis, which can narrow or block arteries and lead to symptoms such as leg pain with walking or poor wound healing [34]. The possible impact of PAD on great toe FM parameters was assessed in 50 patients with mild and 25 with severe PAD and compared to 50 healthy controls [35]. FM relative amplitudes did not differ among groups, with median values ranging from 17.6% to 19.5% of the average LDF value. Contrastingly, the FM median frequency in patients with severe PAD (0.067 Hz) exceeded that in either mild PAD (0.028 Hz) or in control subjects (0.026 Hz), p < 0.001. The higher FM rate may serve as a compensatory mechanism in response to the reduced arterial blood flow [35]. A different pattern was observed in 23 patients with severe PAD, 9 of whom also had edema [36]. Two frequency bands were defined: a low-frequency band (LF, 0.01-0.15 Hz) and a high-frequency band (HF, 0.15-0.40 Hz). HF waves were absent in healthy controls but consistently present in both limbs of all PAD patients, regardless of edema. LF waves were observed in all groups. These findings suggest that HF FM waves are a distinct feature of severe ischemia, which may help stimulate microvascular activity in severely ischemic tissue [36]. The effects of revascularization on 13 patients with severe PAD were assessed via LDF on the foot dorsum before and 30 days after the procedure [37]. FM was assessed across four frequency bands: cardiac, respiratory, myogenic, and sympathetic neurogenic. After revascularization, the absolute amplitude (p = 0.002) and energy (p = 0.004) of the cardiac component increased significantly. In contrast, no significant changes were observed in the absolute or relative amplitude or energy of the other components. These findings suggest that cardiac-related FM improves following revascularization, while other components remain unchanged [37]. 

Similarly, another study evaluated FM in 15 patients with severe PAD before and after revascularization [38]. LDF was measured on the dorsum of the affected foot with the patient supine. FM was assessed across six physiological frequency bands: cardiac (1-2 Hz), respiratory (≈ 0.2 Hz), myogenic (≈ 0.1 Hz), sympathetic (≈ 0.04 Hz), and two endothelial intervals (NO-dependent ≈ 0.01 Hz and NO-independent ≈ 0.007 Hz). Two weeks after revascularization, normalized wavelet power increased significantly by 49.8% (p = 0.0341) in a narrow band near the myogenic-sympathetic transition (0.050-0.053 Hz), while power decreased by 77.1% (p = 0.0179) in the endothelial range. No changes were observed in the cardiac or respiratory components. The author suggests that revascularization may reduce the local endothelial influence on FM and enhance contributions from centrally regulated mechanisms, such as sympathetic activity [38].

Chronic Venous Insufficiency (CVI)

CVI is a condition where the veins in the lower limbs cannot effectively return blood to the heart, often due to damaged valves or prolonged venous HTN. Over time, this can lead to symptoms such as leg swelling, skin changes, and, in more advanced cases, venous ulcers [39]. In a study that included 43 patients with lipodermatosclerosis (LDS), 14 with varicose veins (VV), and 44 healthy controls, baseline LDF was measured for 5 minutes at 2 cm above the medial malleolus [40]. There was a wide range of vasomotion frequencies reported for each group, with the LDS patients having the greatest range (0.025 to 0.112 Hz) and a median of about 0.050 Hz, which was significantly greater than that of controls (0.040 Hz; p < 0.0007). 

Hypercholesterolemia

Hypercholesterolemia is caused by a combination of genetic factors, lifestyle choices, and diet, and the condition is characterized by elevated LDL or reduced HDL levels, which can lead to vascular and cardiovascular dysfunction [41]. Skin FM in 15 patients with hypercholesterolemia but without clinically apparent arterial disease was compared to 15 age-matched healthy controls, with LDF measurements done for 20 minutes at the dorsal aspect of the middle finger, followed by 20 minutes during acetylcholine (ACh) iontophoresis [42]. At baseline, there were no significant differences in spectral power across any of the five FM bands (cardiac, respiratory, myogenic, neurogenic, and endothelial). However, following ACh iontophoresis, the hypercholesterolemic group showed a significantly lower increase in PSD in the endothelial-related band (0.01-0.02 Hz) compared to controls (p < 0.005) despite exhibiting a similar increase in skin perfusion during ACh iontophoresis. No significant differences in the PSD response were found in the other frequency intervals between groups. The authors conclude that these findings suggest that spectral analysis of FM may detect microvascular endothelial dysfunction in hypercholesterolemic patients and that statin therapy may contribute to functional improvement [42]. 

Diabetes-Related Conditions

Metabolic and endocrine diseases are associated with significant microvascular complications that can disrupt normal FM patterns. In these conditions, endothelial dysfunction, oxidative stress, and impaired autonomic regulation are key contributors to altered vasomotion [43]. Diabetes mellitus (DM) is a metabolic disorder characterized by either autoimmune destruction of pancreatic islet cells (type 1, T1DM) or insulin resistance (type 2, T2DM), leading to elevated blood glucose levels. Prolonged high blood sugar can damage blood vessels, resulting in microvascular complications such as peripheral neuropathy [44]. 

Several researchers have investigated FM-related aspects associated with type 1 DM. One study examined microvascular abnormalities in 17 patients with T1DM and compared them to 40 healthy controls using LDF [45]. LDF measurements were taken on the foot dorsum for four minutes with the patient in a supine position. There was no statistical difference in mean LDF between the groups. However, spectral analysis showed a significantly reduced power in the low-frequency band (0.012-0.045 Hz) in the T1DM group, indicating impaired endothelial and neurogenic activity. The total wavelet energy of LDF oscillations was significantly lower in the T1DM group than in healthy controls (p < 0.01), suggesting an overall reduction in FM activity. The authors speculate that FM may help in diagnosing T1DM. 

Additional information was provided by a study that assessed FM via LDF of the great toe pulp in T1DM patients under resting and post-ischemic conditions [46]. Included were 25 males with T1DM and 13 age- and BMI-matched male controls, all with a normal ankle-brachial pressure index of 1.0-1.2. Following a 20-minute rest period to achieve a steady-state baseline, a three-minute ischemic challenge was applied using a tourniquet inflated to 70 mmHg above ankle systolic pressure, after which the hyperemic response was recorded. At baseline, the median frequency of resting FM was significantly higher in the diabetic group compared to controls (0.133 Hz vs. 0.083 Hz, p < 0.0001), with this difference becoming more pronounced during the post-ischemic phase (0.183 Hz vs. 0.100 Hz, p < 0.05).

Other findings linking FM changes to the diabetic condition were reported in a study that evaluated FM in 40 patients with T1DM using LDF measurements on the forearm, compared with 50 age- and sex-matched healthy controls, at baseline and after a three-minute bicep cuff occlusion [47]. The PSD was used to assess baseline and post-ischemic responses. Their results indicated essentially no differences in PSD between groups during a 20-minute baseline interval across most frequency bands. After occlusion release, both groups showed an increase in PSD across all bands, but the percentage increase in endothelial (0.009-0.02 Hz) and sympathetic-related (0.021-0.060 Hz) bands was significantly lower in the T1DM group (p < 0.001).

The relationship between glucose metabolism and LDF-determined FM measured on the wrist for 25 minutes was evaluated in a large population-based study of over 7,000 participants [48]. Endothelial, neurogenic, and myogenic components of FM across individuals with normal glucose metabolism, prediabetes, and T2DM were evaluated using PSD assessments. After adjustment for confounding variables, HbA1c was inversely and significantly associated with all three FM components. These findings suggest that hyperglycemia may impair multiple components of skin microvascular FM. 

Peripheral Neuropathy

The possible effect of neuropathy on FM in patients with DM was evaluated using dorsal foot and medial malleolus LDF measurements to determine spectral power in each of six FM frequency bands, with the patient’s legs supine and then with one leg in a dependent position [49]. 

The study included 16 healthy controls, 22 with obesity, 15 with T2DM without neuropathy, 13 with subclinical neuropathy, and 16 with confirmed neuropathy. At rest, with legs horizontal, there was no difference in total spectral power between groups. With leg dependency, total spectral power increased (p < 0.001) in all groups with no difference between them. The relative contribution of NO-independent (0.005-0.0095 Hz) and NO-dependent (0.0095-0.021 Hz) endothelial bands increased (p < 0.01), while myogenic components (0.052-0.145 Hz) decreased (p < 0.01). The role of neuropathy on FM parameters is unclear based on these findings. However, a different study that included a neuropathy assessment evaluated 20 healthy controls and 20 age-matched diabetic patients, including eight with T1DM and 12 with T2DM [50]. LDF was measured for five minutes on the pulp of the left index finger. Impaired vasomotion, which was defined as an amplitude of less than one-third that of a matched control, was present in 75% of diabetic subjects. Among six tests of neuropathy, only the warm thermal sensory threshold showed a significant correlation with FM amplitude; thus, based on these findings, neuropathy appears to be a modifier of FM in persons with this condition. 

A further study exploring the role of diabetic related neuropathy compared T1DM patients with and without peripheral neuropathy to healthy controls [51]. There were 12 with neuropathy (T1DM+N), 10 without neuropathy (T1DM-N), and 10 age-matched non-diabetic controls. LDF was measured on the dorsum of both hands and both feet for 10 minutes. The FM frequency and amplitude were all similar across all groups. However, the T1DM+N group had a significantly lower relative amplitude in the feet compared to both the T1DM-N group (7.2% vs. 13.5%, p = 0.012) and the control group (7.2% vs. 10.3%, p = 0.016). This reduction in amplitude was localized to the feet, consistent with the distal presentation of diabetic neuropathy. This study’s findings provide some additional support for the potential role of peripheral neuropathy as an FM modifier under resting conditions. 

Further support comes from a study that evaluated exercise effects in patients with T2DM and peripheral neuropathy [52]. Eighty-six participants were divided into five groups: 16 healthy controls, 22 individuals with obesity, 16 T2DM without neuropathy (T2DM-N), 15 with subclinical neuropathy (T2DM+SCN), and 17 with confirmed neuropathy (T2DM+N). After a six-minute walking test, LDF was recorded on the dorsal right foot for 10 minutes while participants rested in the supine position. Spectral analysis revealed that total power increased in all groups during recovery, but this increase was significantly blunted in the T2DM+N group (p < 0.05). The relative contribution of endothelial FM components increased post-exercise in all groups but was significantly lower in the T2DM+N group (p < 0.01). The FM neurogenic component decreased during recovery in all groups, though this decline was less pronounced in the T2DM+N group (p = 0.05). The myogenic component decreased overall, with lower values seen in the obesity and T2DM-N group. These findings suggest that peripheral neuropathy impairs post-exercise FM responses, particularly those involving endothelial and neurogenic mechanisms [52]. 

The potential impact of forearm and foot dorsum skin heating on FM in patients with diabetic neuropathy is another, as that was investigated by heating skin to 44°C with and without phenylephrine iontophoresis [53]. The study included 10 healthy controls, 11 T2DM patients without neuropathy (T2DM), 11 with peripheral neuropathy (T2DM+PN), and nine with combined autonomic and peripheral neuropathy (T2DM+AN). In controls and the T2DM group, the combination of phenylephrine and warming produced structured rhythmic FM around 0.1 Hz. In contrast, FM in the T2DM+PN and T2DM+AN groups appeared more irregular and disorganized, suggesting a loss of the normal rhythmic pattern seen in healthy individuals. The potential significance of this 0.1 Hz in patients with diabetes was subsequently investigated in 20 T1DM and 22 T2DM patients [54]. Capillary RBC velocity (CBV) was measured in single capillaries of the dorsal middle phalanx of the left ring finger using laser Doppler anemometry. Impaired FM was defined by the authors as peak-to-peak FM amplitudes more than two standard deviations below the mean of previously measured normal subjects. According to this definition, impaired vasomotion was detected in 90% of T1DM patients with abnormal cardiac autonomic function and in 40% with normal cardiac autonomic function (p = 0.029). In the T2DM group, the FM amplitude did not differ significantly between patients with or without autonomic neuropathy. These findings are consistent with the concept that a reduced FM at ≈ 0.1 Hz may be an early indicator of sympathetic dysfunction in diabetic patients, especially those with T1DM [54].

In sum, diabetic peripheral neuropathy was associated with reduced vasomotion amplitude, particularly at 0.1 Hz, diminished endothelial and neurogenic FM responses following exercise or thermal stimulation, and a loss of rhythmic organization in spectral activity. These impairments were often detectable despite preserved perfusion or normal autonomic testing, further highlighting the value of FM analysis in detecting subtle microvascular dysfunction.

Autonomic Neuropathy

In addition to studies assessing peripheral FM in diabetic neuropathy, one study evaluated whether autonomic-related vasomotor impairments in diabetic neuropathy could be reversed with frequency-rhythmic electrical modulation system (FREMS) therapy. The study included 10 patients with T2DM and confirmed dysautonomia polyneuropathy and 10 healthy controls. LDF measurements were taken on the volar forearm, with recordings taken for six minutes before, during, and after FREMS stimulation, which was applied in three sessions during a single 30-minute protocol. Prior to stimulation, the diabetic group showed significantly reduced vasomotion at 0.1 Hz compared to controls. During FREMS, the 0.1 Hz vasomotion power spectrum increased by approximately 50% in the diabetic group (p < 0.05), while no significant change occurred in healthy controls. This enhancement in 0.1 Hz power remained elevated in the diabetic group (p < 0.05) immediately following the intervention, suggesting that FREMS may partially and temporarily restore 0.1 Hz vasomotion in dysautonomic diabetic patients [55]. Other aspects of the reduced 0.1 Hz power have been studied in patients with T1DM [56]. 

Further studies examining the impact of autonomic neuropathy compared FM features between 25 T2DM patients with autonomic neuropathy and 36 healthy age-matched controls [57]. LDF values were obtained at the upper extremity (caput ulnae) and lower extremity (medial malleolus) bilaterally for 30 minutes and analyzed via wavelet methods. Despite using high-resolution wavelet analysis, no individual frequency band consistently differed between groups, suggesting that baseline measurements alone may not be sensitive enough to detect early autonomic changes [57]. However, a possible link between sudomotor dysfunction and FM features has been reported [58]. In this study, 68 T2DM patients with varying degrees of foot skin impairment were compared with 25 healthy controls using wavelet analysis of LDF measured for 20 minutes on the great toe dorsum. Results indicated that patients with absent sympathetic skin response and pre-ulcerative skin changes had the lowest total spectral amplitude and significantly reduced endothelial and neurogenic FM components (p < 0.05-0.01). These findings suggest that diminished low-frequency FM may reflect early microvascular dysfunction in diabetic neuropathy, before clinically obvious nerve and vascular impairment appear. 

A related study investigated whether baseline FM differed across T2DM patients with different stages of diabetic neuropathy [59]. Included were 24 patients with clinical neuropathy, 27 with subclinical neuropathy, 26 without neuropathy, and 25 healthy controls. LDF was measured at the dorsum of the right big toe for 20 minutes, and spectral power in the endothelial, neurogenic, and myogenic frequency bands was determined. The clinical neuropathy group had significantly reduced power in the endothelial and neurogenic bands compared to the control group (p < 0.01). This study’s overall findings may indicate that spectral FM analysis of endothelial and neurogenic components may help identify early microvascular dysfunction in diabetic patients before neuropathy becomes clinically relevant. 

Charcot Neuroarthropathy

Charcot foot, a destructive joint disorder, can develop from uncontrolled diabetes, resulting in distal peripheral neuropathy [60]. To explore this relationship, foot dorsum LDF heating responses (35°C to 45°C) in diabetic patients with Charcot arthropathy were compared with those of 11 healthy controls [61]. Nine patients had diabetic neuropathy and Charcot arthropathy (D+C), and 12 had diabetic neuropathy without Charcot disease (D-C). At 35°C, no FM spectral differences among groups were found. However, at 45°C, although FM significantly increased in the D+C and control groups, it was blunted in the D-C group (p < 0.02). The investigators suggest that the findings may indicate that, whereas patients with Charcot arthropathy retain some vasomotor responsiveness despite neuropathy, the reduced FM seen in those without Charcot disease could be a protective mechanism that limits excessive blood flow and bone turnover [61].

Across the reviewed studies, diabetes was associated with reduced spectral power in the endothelial and neurogenic bands, altered myogenic regulation, and blunted FM responses to thermal, ischemic, or postural challenges. These changes were often detectable before clinical signs of neuropathy, supporting the use of spectral analysis in identifying early microvascular dysfunction in diabetic patients.

Chronic Kidney Disease (CKD)

CKD is a progressive condition characterized by a gradual loss of kidney function over time. It is typically defined by a decreased glomerular filtration rate (GFR) of less than 60 mL/min/1.73 m² or evidence of kidney damage lasting more than three months [62]. One study assessed whether microvascular FM is altered in patients with stage III-IV CKD, defined as creatinine clearance 15-59 mL/min [63]. The study included 32 patients with CKD and 32 age- and sex-matched controls. LDF was measured on the medial forearm for five minutes, followed by three minutes of ischemia by cuff inflation and a 10-minute post-occlusion recovery. FM spectral analysis indicated that CKD was associated with a reduced (p < 0.05) relative PSD of the myogenic component (0.06-0.2 Hz) under basal and post-ischemic conditions. 

Spinal Cord Injury (SCI)

SCI is a neurological condition characterized by damage to the spinal cord from either traumatic or non-traumatic causes. The injury typically begins with a primary mechanical insult, followed by secondary processes like inflammation, ischemia, and oxidative stress, which can further damage both the nervous and vascular systems over time [64]. 

The impact of varying levels of physical activity on the regularity of skin FM patterns in persons with SCI was examined using multiscale sample entropy (MSE) [65]. The study included 37 participants divided into three groups: 12 athletes with SCI (ASCI), nine sedentary individuals with SCI (SSCI), and 16 able-bodied controls (ABC). Sacral skin blood flow was measured using LDF for 10 minutes, followed by 50 minutes of local heating to 42°C. Across all groups, FM became more regular during heating, but the SSCI group showed significantly lower MSE values compared to the ASCI and AB groups (p < 0.05). Wavelet analysis confirmed that the SSCI group had lower cardiac and myogenic amplitudes. The authors concluded that these findings suggest that physical activity may help maintain the complexity and regulation of FM after SCI [65]. 

A somewhat related study investigated whether basal sympathetic nerve activity persisted in the skin microcirculation of individuals with complete tetraplegia [66]. The study included 10 male patients with traumatic complete tetraplegia and 10 age-matched healthy male controls. With the patient supine, 30-minute LDF recordings were obtained simultaneously at four sites on the lower limb: the plantar surface of the first toe, the medial malleolus, the dorsum of the foot, and the lateral calf. Wavelet transform analysis revealed that total spectral power did not differ significantly between groups at any site. However, individuals with tetraplegia had significantly reduced neurogenic activity (-23 to -48% reduction), myogenic activity (-39 to -62% reduction), and endothelial activity (-21 to -54% for NO-dependent and -16 to -43% for non-NO-dependent components) compared to controls. In contrast, cardiac-related FM was 25-68% higher in the tetraplegia group, suggesting a mechanism to compensate for impaired vascular regulation. These findings suggest that FM regulation in the neurogenic and endothelial bands is substantially altered in complete tetraplegia [66]. 

A separate study assessed whether the nonlinear complexity of skin FM differs between individuals with SCI and nondisabled controls using an analytical method called multifractal detrended fluctuation analysis (MDFA) [67]. The study included 23 participants: four with complete SCI, seven with incomplete SCI, and 12 nondisabled controls. Skin perfusion was measured using LDF on the sacral skin while patients lay prone. The protocol included a 10-minute preheating phase, 50 minutes of local heating to 42°C, and a 10-minute post-heating period. The main result indicated that the FM complexity of the metabolic band (0.0095-0.02 Hz) and the neurogenic band (0.021-0.05 Hz) in individuals with complete and incomplete SCI is lower than in non-disabled persons during maximal vasodilation. However, it is unclear how the complexity feature relates to physiology, though it may prove useful. 

Another study assessed sacral and gluteus muscle skin FM, which are anatomical areas commonly at risk for pressure sores, by comparing 10 young healthy adults, 20 healthy elderly participants, and 20 individuals with SCI [68]. SCI participants were further subdivided into 15 patients with distinct FM and five without detectable sacral FM. LDF was measured for 3 minutes at baseline, 3 minutes during arterial occlusion with 400 mmHg of external pressure, and 9 minutes during the post-occlusion hyperemia. In the 15 SCI patients with FM, the spectral power of resting FM was significantly greater (p < 0.05) than in all other groups, without a difference among groups in the FM central frequency that was approximately 0.155 Hz. The combined findings of this study suggest that patients without visible sacral FM may have impaired microvascular reactivity, which may contribute to the development of pressure sores. 

Schizophrenia

Schizophrenia is a psychiatric disorder that affects how someone perceives reality, often leading to symptoms like hallucinations, delusions, and disorganized thinking. It typically develops in late adolescence or early adulthood and can significantly disrupt daily functioning. While researchers believe that a mix of genetic, environmental, and neurodevelopmental factors plays a role in its pathogenesis, no single cause has been identified [69]. 

One study assessed peripheral microvascular function in 21 unmedicated patients with acute paranoid schizophrenia compared to 21 healthy controls [70]. Volar forearm LDF was measured during a 15-minute baseline followed by a three-minute occlusion and a 15-minute post-occlusion LDF response. FM was analyzed using the PSD across five frequency bands defined as due to endothelial activity (0.009-0.02 Hz), sympathetic activity (0.020-0.06 Hz), myogenic activity (0.06-0.20 Hz), respiratory activity (0.20-0.6 Hz), and cardiac activity (0.60-1.6 Hz). With respect to the FM analysis, power across all frequency bands tended to be higher in the patient group, but only the resting respiratory power was significantly higher (p < 0.005).

Autoimmune and Inflammatory Conditions 

Autoimmune and inflammatory diseases, including systemic sclerosis (SS) and other rheumatic conditions, are often associated with microvascular complications driven by chronic inflammation, immune-mediated endothelial dysfunction, and progressive tissue remodeling. These processes can disrupt vasomotor control and alter FM dynamics across multiple frequency components.

Systemic Sclerosis

Scleroderma is a connective tissue disorder divided into two main subtypes: localized scleroderma (LS) and SS [71]. SS is further classified into limited and diffuse subtypes, with the diffuse form characterized by more extensive skin thickening and internal organ damage than the limited type. In contrast, LS primarily affects the skin and subcutaneous tissue without involving internal organs [71]. The effects of SS on finger dorsal skin FM were evaluated in 26 patients and 20 age-matched healthy controls using LDF combined with the iontophoresis of vasodilators Ach and SNP [72]. LDF was measured during a 10-minute baseline and a 10-minute steady-state phase following each vasodilator. Compared with controls, SS patients showed significantly lower baseline activity in the myogenic band (p < 0.00005) and, after Ach, reduced FM power in the endothelial (p < 0.005), sympathetic (p < 0.005), and myogenic bands (p < 0.005). Following SNP iontophoresis, lower FM responses were observed in the endothelial (p < 0.01), sympathetic (p < 0.05), and myogenic bands (p < 0.05). These findings indicate that SS is associated with impaired FM across multiple bands. A follow-up study assessed changes in FM after 10 weeks of simvastatin therapy in 13 female patients with SS and mild hypercholesterolemia, compared to 15 female controls. LDF measurements were taken on the finger dorsum to obtain a 10-minute rest value, followed by three-minute finger occlusion and release. These were performed before treatment and after 10 weeks. Before treatment, the SS group showed no post-ischemic increase in FM power in any frequency band, whereas healthy controls exhibited significant increases in endothelial, sympathetic, myogenic, respiratory, and cardiac bands (p < 0.05 to p < 0.001). After 10 weeks of simvastatin therapy, the SS group demonstrated a significant post-ischemic increase in the myogenic (p < 0.01) and cardiac (p < 0.01) bands, indicating partial restoration of FM function. The improvements observed after simvastatin therapy suggest that targeting microvascular tone, tone-particularly in the myogenic and cardiac components, may help enhance FM responses and vascular reactivity in patients with SS [73]. 

Rheumatic Disease

Rheumatic diseases are a group of chronic conditions that primarily affect the joints and connective tissues, often driven by inflammation and abnormal immune responses. Over time, they can lead to pain, swelling, organ involvement, and long-term disability if not properly managed. Among them, osteoarthritis (OA) and rheumatoid arthritis (RA) are the most common, with knee OA affecting up to 10% of adults by age 74, and RA affecting nearly 0.5-1% of adults worldwide [74].

One study assessed FM changes in 60 patients with rheumatic diseases compared to 32 healthy controls using LDF and tissue oximetry during a cold pressor test [75]. LDF was measured on the middle finger pulp before, during, and after five minutes of cold-water immersion at 15°C. At baseline, patients with rheumatic disease showed significantly higher LDF and greater power in the respiratory and cardiac bands compared to controls (p < 0.01). During cooling, healthy controls exhibited a stronger vasoconstrictive response, while patients had reduced perfusion decreases and increased spectral power in the respiratory and cardiac bands. When post-cooling perfusion and cardiac band amplitude were combined, researchers distinguished patients from healthy controls with 92% sensitivity, 97% specificity, and an area under the curve of 0.92, supporting the diagnostic value of FM-based metrics for identifying microvascular dysfunction in rheumatic diseases. Therefore, these findings indicate that patients with rheumatic disease display FM patterns that markedly differ from those of healthy individuals, suggesting issues with microvascular function. They tend to have higher baseline blood flow, a less pronounced vasoconstrictive response to cold, stronger respiratory and cardiac oscillations, increased myogenic tone, and lower oxygen consumption during recovery.

Asthma

Asthma is a chronic respiratory condition marked by inflammation and narrowing of the airways [76]. This often causes episodes of wheezing, coughing, and shortness of breath. These symptoms may result from an overactive immune response to various triggers, such as allergens, respiratory infections, pollutants, or stress. To determine whether skin FM was altered in patients with asthma, forearm LDF was measured in 11 patients with a forced expiratory volume in one second (FEV1) below 80% and nine patients with FEV1 greater than 80% [77]. LDF was measured at rest, followed by three minutes of upper arm ischemia induced by cuff inflation, and six minutes of post-occlusive hyperemia. Compared with patients in the high FEV1 group, those in the low FEV1 group had lower FM amplitude in the myogenic, neurogenic, and endothelial frequency bands, with reductions ranging from 40 to 52 % (p < 0.05). No significant differences in average or peak skin blood perfusion during reactive hyperemia were found between groups, suggesting that microvascular tone regulation is impaired in the low FEV1 group despite preserved perfusion [77]. 

Leprosy

Leprosy, also called Hansen’s disease, is a long-lasting infectious disease caused by Mycobacterium leprae that mainly affects the skin and peripheral nerves. It is typically categorized into two main forms based on the host’s immune response: tuberculoid leprosy, characterized by a strong immune response and few lesions, and lepromatous leprosy, characterized by a weak immune response, widespread skin involvement, and a higher bacterial load [78]. 

One study assessed microvascular changes in seven patients with tuberculoid leprosy using LDF to compare cutaneous plaques with symmetrical healthy skin on the contralateral limb [79]. LDF was measured for 20 minutes at the center of each lesion and at anatomically matched control sites. FM amplitudes showed no significant differences between sites in any of the five standard frequency bands. Another study evaluated FM in 10 men with lepromatous leprosy and 10 age- and sex-matched controls using LDF [80]. LDF was measured for 20 minutes at the wrist dorsum, and FM was assessed using spectral analysis across five frequency bands. No significant differences in FM amplitude were observed between groups across the endothelial, neurogenic, myogenic, respiratory, or cardiac components, indicating that any microvascular dysfunction in this condition does not involve changes in FM. The key takeaway is that in leprosy, studies show that baseline FM is largely preserved, with no significant differences in oscillatory components or flow distribution compared to healthy controls.

Table 2 summarizes the key FM findings.

**Table 2:** Summary of key flowmotion findings This table summarizes the main findings from each study included in this scoping review. The columns are organized by reference numbers, subjects, the anatomical sites, the laser Doppler flow protocol, and the primary flowmotion (FM) findings. Note that some reference numbers are omitted, as they were included for context only.

Reference No.	Subjects	Measured Site	Laser Doppler Flow (LDF) Protocol	Main Flowmotion (FM) Findings
[26]	59 hypertensive men vs. 30 controls	Lateral forearm	LDF for 10 minutes. Wavelet analysis to assess frequency bands. Endothelial (0.0095-0.02 Hz), Neurogenic (0.021-0.052 Hz), Myogenic (0.052-0.145 Hz), Respiratory (0.145-0.6 Hz), and cardiac (0.6-2.0 Hz).	Hypertensives had decreased neurogenic, myogenic, and cardiac amplitudes (p < 0.05). No change in others.
[27]	18 hypertensives vs. 18 age-matched controls	Ventral forearm	LDF for 20 minutes at 33°C and during local heating to 42°C. Wavelet analysis to assess endothelial (0.0095-0.02 Hz), neurogenic (0.02-0.05 Hz), and myogenic (0.05-0.15 Hz) bands.	At 33 °C, hypertensives had reduced neurogenic activity (p < 0.01). During heating, myogenic activity increased (p = 0.04).
[28]	31 hypertensives, 17 normotensives without a family history of hypertension, 22 normotensives with a family history of hypertension	Forearm	LDF was measured when the skin was rapidly heated to 44 °C and maintained for 8 minutes.	FM changes only in normotensives with a family history of hypertension. Myogenic, Respiratory, and Cardiac bands were reduced.
[29]	46 newly diagnosed, hypertensives vs. 20 normotensives	Forearm	LDF while resting for 30 minutes.	Hypertensives had higher spectral amplitudes across most frequency bands, but the myogenic relative contribution was less (p < 0.01).
[30]	63 newly diagnosed hypertensives divided into two subgroups vs.30 normotensive controls.	Forearm	LDF at rest was measured for six minutes. Wavelet analysis is used for spectral FM power.	One hypertensive subgroup had reduced spectral amplitudes in the endothelial and neurogenic bands (p < 0.002). Another subgroup had an increased respiratory amplitude (p < 0.004), and myogenic spectral power (p < 0.05).
[31]	29 healthy young adults, 22 healthy older adults, and 22 older adults with treated hypertension	Wrist	30-minute recordings of LDF, electrocardiogram, and respiratory effort at rest.	Hypertension was associated with reduced spectral power in the neurogenic and myogenic frequency bands
[33]	61 patients with Raynaud’s phenomenon and 17 controls. Patients were divided into three groups based on the dominant frequency identified in their resting baseline FM power spectra.	The palmar surface of both index fingers	LDF for 20 minutes, then heated to 40 °C, then cooled to 5°C.	Controls had robust low-frequency FM. Raynaud's patients had low-frequency components that were significantly lower than in controls.
[35]	50 healthy controls 50 patients with mild peripheral arterial disease (PAD) 25 with severe PAD	Palmar thumb and plantar great toe	LDF was measured for 5 minutes with patients at rest and in the supine position.	FM between 1-4 cycles/min was in 12% of control toes and 19% of fingers, in 46% of toes in mild PAD, and in 77% in severe PAD. The median FM frequency increased from 1.6 in controls to 4.0 in severe PAD (p < 0.001).
[36]	14 patients with PAD and edema 9 with PAD without edema, and 10 healthy controls).	Ischemic and contralateral leg at the great toe pulp, and on the ischemic leg’s 2nd metatarsal, and the ankle	LDF was with the patient supine, then repeated after passively lowering the ischemic limb below heart level by flexing the knee. Recordings were taken at each position for 3 minutes, with the final 2.5 minutes of each used for fast Fourier transform (FFT) analysis of low-frequency (LF, 0.01-0.15 Hz) and high-frequency (HF, 0.15-0.40 Hz) oscillations.	HF waves are absent in controls but present bilaterally in all PAD patients. LF and HF power were lower in ischemic legs vs. contralateral and control legs (p < 0.001). After lowering the ischemic leg, LF power (p < 0.005) and HF power (p < 0.001) increased. In healthy legs, LF power decreased at the toe (p < 0.05). FM frequency among groups or positions were not different
[37]	13 patients with severe PAD before and after revascularization	Foot dorsum	LDF was measured at the foot dorsum while supine for 20 minutes, one week before and within 30 days after revascularization. FM was assessed in respiratory, cardiac, myogenic, and sympathetic bands.	After revascularization, the cardiac component amplitude (p = 0.002) and energy (p = 0.004) increased. No significant changes were observed in the other components.
[38]	15 patients with severe PAD before and after revascularization	Foot dorsum	FM was assessed across six frequency bands: cardiac, respiratory, myogenic, sympathetic, and two endothelial intervals (NO-dependent and NO-independent).	After revascularization, power increased by 49.8% (p = 0.0341) in a band near the myogenic-sympathetic transition (0.050-0.053 Hz), while power decreased by 77.1% (p = 0.0179) in the endothelial band.
[40]	43 patients with lipodermatosclerosis (LS) vs. 14 with varicose veins (VV) vs. 44 healthy controls	Two cm above the medial malleolus	LDF was measured for five minutes. In a subset of patients, the area was heated to 43°C while LDF changes were monitored.	Compared to controls, the LS group had a greater baseline FM amplitude and frequency. After heating, FM amplitude and frequency increased in controls and the VV group but not in the LS group.
[42]	15 patients with high cholesterol without clinical arterial disease vs. 15 age-matched healthy controls	Dorsal aspect of the middle finger	Baseline measurements were done for 20 minutes, followed by 20 minutes during acetylcholine iontophoresis (AI). Spectral analysis was done of FM cardiac, respiratory, myogenic, neurogenic, and endothelial bands.	At baseline, no differences in spectral power across any FM band. After AI, patients had a smaller increase in endothelial-related band power (0.01-0.02 Hz) vs. controls (p < 0.005).
[45]	17 patients with type 1 diabetes mellitus vs. 40 healthy controls	Foot dorsum	LDF measurements were done for four minutes with the patients and subjects supine.	Patients had reduced power in the low-frequency band (0.012-0.045 Hz) and reduced total power compared to healthy controls (p < 0.01), suggesting an overall reduction in FM activity.
[46]	25 males with type 1 diabetes with normal ankle-brachial index (1.0-1.2) vs. 13 controls	Great toe pulp	LDF was measured at a skin temperature of 30°C for 20 minutes. A 3-minute ischemic challenge and post-occlusion hyperemia were produced by cuff inflation and then release.	Pre-ischemic FM in patients was higher than in controls (p < 0.0001). The difference was greater during the post-ischemic phase.
[47]	40 patients with type 1 diabetes mellitus vs. 50 healthy controls	Medial forearm	LDF was measured for 20 minutes at baseline, followed by a 3-minute ischemic period and a 10-minute post-occlusion hyperemic period using cuff occlusion and release.	In patients, the baseline power of the cardiac frequency band was higher (p = 0.025), but no difference in other bands. During hyperemia, FM increased in both groups, but the percentage increase in endothelial- and sympathetic-related FM was less in patients (p < 0.001).
[48]	7293 participants of a large cohort with normal glucose metabolism (NGM), or prediabetes (PreD), or type 2 diabetes.	Wrist dorsum	LDF was measured for 25 minutes under standardized conditions.	After adjustment for confounding variables, HbA1c was found to be significantly and inversely associated with three FM components, regardless of glucose metabolism status as measured by power spectral density.
[49]	16 healthy controls, 22 with obesity, 15 with type 2 diabetes 13 with subclinical neuropathy 16 with neuropathy.	Foot dorsum	LDF was measured with the subject supine. Power in each of six FM frequency bands was determined with the patient’s legs supine and then with one leg in a dependent position	Spectral analysis of FM during the leg dependency phase showed that total spectral power increased at the medial malleus (p < 0.001), with no difference between groups.
[50]	20 healthy controls vs. 20 age-matched diabetic patients (8 with type 1 and 12 with type 2 diabetes)	Index finger pulp	LDF was measured at rest for 5 minutes. Low-frequency FM power was determined via fast Fourier transform.	Patients had reduced FM amplitude vs. controls (24.9 ± 6.4 vs. 129.0 ± 33.2, p = 0.0039). Impaired FM, defined as an amplitude of < 1/3 that of a matched control, was present in 75% of patients.
[51]	12 diabetic patients with neuropathy (T1DM+N) vs. 10 diabetic patients without neuropathy (T1DM-N) vs. 10 age-matched non-diabetic controls	Four sites on the dorsum of both hands and feet	LDF was measured at rest simultaneously for 10 minutes per site.	The frequency and amplitude of FM were similar in the hands for all groups, and FM frequency in the feet did not differ. The T1DM+N group had a lower relative amplitude in the feet compared to both the T1DM-N group (7.2% vs. 13.5%, p = 0.012) and the control group (7.2% vs. 10.3%, p = 0.016).
[52]	16 healthy controls vs. 22 with obesity 16 type 2 diabetes without neuropathy (T2DM-N) 15 with subclinical neuropathy T2DM+SCN) 17 with neuropathy (T2DM+N).	Foot dorsum	LDF was recorded on the dorsal right foot for 10 minutes, after a six-minute walking test, while participants rested in the supine position.	Total spectral power increased in all groups during recovery, but the increase was blunted in the T2DM+N group (p < 0.05). The contribution of endothelial FM components increased post-exercise in all groups, but values were lower in the T2DM+N group (p < 0.01).
[53]	10 healthy controls 11 type 2 diabetes (no neuropathy (T2DM-N) vs. 11 with peripheral neuropathy (T2DM+PN) vs. 9 with combined autonomic and peripheral neuropathy (T2DM+AN).	Left foot dorsum Left forearm	LDF was measured at baseline, during local heating to 44°C, and during heating combined with phenylephrine iontophoresis.	In controls and the T2DM-N group, the combination of phenylephrine and warming produced structured rhythmic FM around 0.1 Hz. In contrast, FM in the T2DM+PN and T2DM+AN groups appeared more irregular and disorganized, suggesting a loss of regular rhythmic pattern seen in healthy individuals.
[54]	20 patients with type 1 diabetes mellitus vs. 22 with type 2 diabetes mellitus, 29 ± 2 years vs. 62 ± 2 years).	Ring finger dorsum.	Capillary blood cell velocity was measured in single nailfold capillaries using laser Doppler anemometry.	The authors defined impaired FM as peak-to-peak FM amplitudes > two standard deviations below the mean of previously measured normal subjects. Accordingly, impaired FM was in 90% of type 1 diabetes patients who had abnormal autonomic function and in 40% of type 1 diabetes patients with normal function (p = 0.029). .
[55]	10 patients with type 2 diabetes mellitus and dysautonomia vs. 10 healthy controls	Volar forearm	LDF was measured 6 minutes before, during, and after stimulation, which was applied in three sessions of a single 30-minute protocol.	Before stimulation, the diabetic group showed reduced FM at 0.1 Hz compared to controls. During stimulation, the 0.1 Hz FM spectral power increased by 50% in the diabetic group (p < 0.05), while no change occurred in healthy controls.
[56]	23 patients with type 1 diabetes vs. 23 age-matched healthy controls.	Volar forearm	LDF was continuously measured for 4 minutes during controlled breathing at 20 breaths per minute, and spectral analysis was done to isolate specific frequency bands expressed as cycles per minute.	Thirteen patients (57%) had abnormally reduced low-frequency FM, defined as values > two standard deviations below the control group mean, 2.57 ± 0.16 vs. 3.48 ± 0.09, p < 0.001.
[57]	25 type 2 diabetes patients with autonomic neuropathy 18 neuropathic diabetic patients vs. 36 healthy age-matched controls	Upper extremity (caput ulnae) and lower extremity (medial malleolus) bilateral	LDF values were obtained for 30 minutes, and FM was analyzed via wavelet methods.	Spectral amplitudes tended to be greatest in controls, intermediate in diabetic autonomic neuropathy, and lowest in diabetic neuropathy. But differences were significant only in one arm. Across all groups, FM amplitudes were higher in arms than in legs.
[58]	68 type 2 diabetes patients with varying degrees of foot skin impairment vs. 25 healthy controls	Great toe dorsum	LDF was measured for 20 minutes on the great toe dorsum in all subjects. FM spectrum determined by wavelet analysis	Patients with absent sympathetic skin response or with pre-ulcerative skin changes had reduced total spectral amplitude and reduced endothelial and neurogenic FM (p < 0.05 -0.01)
[59]	24 patients with clinical neuropathy vs. 27 with subclinical neuropathy vs. 26 without vs. 25 age-matched controls	Great toe dorsum	LDF was measured for 20 minutes after 10 minutes of supine resting	The clinical neuropathy group had significantly reduced power in the endothelial and neurogenic bands compared to the control group (p < 0.01).
[61]	9 patients with both diabetic neuropathy and Charcot arthropathy (D+C) vs. 12 with diabetic neuropathy without Charcot disease (D-C) vs. 11 healthy controls	Foot dorsum	LDF was measured for 5 minutes at a skin temperature of 35°C, then at 45°C. FM frequencies were determined using a fast Fourier transform.	At 35°C, FM power was low across all groups, with no significant differences. At 45°C, FM power increased in the D+C and control groups but was blunted in the D-C group (p < 0.02).
63]	32 patients with chronic kidney disease (CKD) vs. 32 age- and sex-matched controls. Patients had stage III-V, without diabetes or other cardiovascular disease.	Forearm medial	LDF was measured for 5 minutes at rest and for 10 minutes after a 3-minute occlusion-release procedure.	The percent increase in power spectral density (PSD) in the endothelial band after ischemia was lower in patients vs. controls (185% ± 98% vs. 279% ± 243%, p < 0.05), and normalized endothelial PSD did not significantly increase in patients. Normalized myogenic PSD was also less in patients at rest and post-ischemia (p < 0.05).
[65]	12 athletes with spinal cord injury (ASCI) vs. nine sedentary individuals with spinal cord injury (SSCI) vs. 16 able-bodied controls.	Sacral Skin	LDF was measured for 10 minutes at baseline and 50 minutes during local heating to 42°C. Multiscale sample entropy was used to assess FM regularity	Across all groups, FM became more regular during heating, but the SSCI group became less regular vs. the ASCI and control groups (p < 0.05). Wavelet analysis indicated that SSCI patients had lower cardiac and myogenic amplitudes during heating.
[66]	10 male patients with traumatic complete tetraplegia vs. 10 age-matched healthy male controls	Great toe pulp, Medial malleolus, Foot dorsum. Lateral calf	LDF was simultaneously measured at the four sites continuously for 30 minutes while participants lay supine. Spectral power density (SPD) assessed via wavelet analysis.	SPD was not different between groups at any site. However, patients vs. controls had significantly reduced neurogenic activity (-23 to -48%), myogenic activity (-39 to -62%), and endothelial activity (-21 to -54% for NO-dependent and -16 to -43% for non-NO-dependent components).
[67]	4 patients with complete spinal cord injury (CSCI) vs. 7 with incomplete spinal cord injury (ISCI) vs. 12 nondisabled controls	Sacral skin	LDF was measured with subjects in the prone position for a 10-minute pre-heating interval, a 50-minute local heating to 42°C, and a 10-minute post-heating period.	CSCI and ISCI groups showed reduced metabolic FM complexity during maximal vasodilation (p < 0.05). Additionally, neurogenic FM complexity decreased during heating in both SCI groups (p < 0.05) and was lower in the CSCI group compared to controls at peak vasodilation (p < 0.05).
[68]	10 healthy young individuals vs. 20 elderly participants vs. 20 spinal cord injury patients (SCI)	Sacrum Gluteus muscle	LDF was measured for 3 minutes at baseline and 9 minutes after a 3-minute blood flow interruption. Patients were subdivided into 15 with distinct FM and five without detectable sacral FM.	At the sacrum, FM power increased during hyperemia in patients who had a resting FM. The SCI patients (N=15) actually had a greater resting FM power than the controls (p < 0.05). A similar pattern was observed at the gluteus.
[70]	21 patients with acute paranoid schizophrenia vs. 21 healthy matched controls.	Forearm volar surface	LDF was measured for 15 minutes at rest and for 15 minutes after the release of a three-minute brachial artery occlusion.	With respect to the FM analysis, power across all frequency bands tended to be higher in the patient group, but only the resting respiratory power was significantly higher (p < 0.005).
[72]	26 patients with systemic sclerosis (SS) vs. 20 age-matched healthy controls	Finger dorsum	LDF was measured during a 10-minute baseline and during 10-minute steady-state phases after iontophoresis of acetylcholine (ACH) or sodium nitroprusside (SN) .	Patients had lower baseline FM activity in the myogenic band (p < 0.00005) and a smaller increase after ACH in the endothelial (p < 0.005), sympathetic (p < 0.005), and myogenic bands (p < 0.005). After SN, SS increases were less in the endothelial (p < 0.01), sympathetic (p < 0.05), and myogenic bands (p < 0.05).
[73]	13 female patients with systemic sclerosis (SS) plus mild hypercholesterolemia were evaluated pre- and post-treatment with 10 weeks of simvastatin vs. 15 healthy female controls.	Finger dorsum	LDF was measured for 10 minutes before and after a three-minute, 200 mmHg arterial occlusion applied via a pneumatic cuff on the same finger.	Patients had no post-ischemic increase in FM across any frequency band, whereas controls exhibited significant increases in endothelial, sympathetic, myogenic, respiratory, and cardiac components (p < 0.05 to p < 0.001). After 10 weeks of treatment, patients showed a significant post-ischemic increase in the myogenic (p < 0.01) and cardiac (p < 0.01) bands.
[75]	60 patients with rheumatic diseases vs. 32 healthy controls	Finger pulp	LDF was measured before, during, and after five minutes of cold-water immersion at 15°C.	At baseline, patients had higher LDF perfusion and greater power in the respiratory and cardiac bands (p < 0.01). During cooling, patients had a blunted LDF decrease and increased spectral power in the respiratory and cardiac bands.
[77]	20 patients with asthma, divided by forced expiratory volume in one second (FEV1) values < 80% (N =11, low) or > 80% (N = 9, high)	Forearm	LDF was measured at rest and for 6 minutes after 3 minutes of upper-arm ischemia induced by cuff inflation.	Patients with FEV1 < 80% had a lower FM power in the myogenic (~52% decrease, p < 0.05), neurogenic (~40% decrease, p < 0.05), and endothelial (~44% at rest, p < 0.05; ~46% during hyperemia, p < 0.05). No significant differences were found in resting LDF or peak LDF values during reactive hyperemia between groups.
[79]	7 male patients with tuberculoid leprosy	Center of each tuberculoid leprosy lesion.	LDF measurements were taken for over 20 minutes at the center of each lesion and at matched control sites free of lesions. Spectral analysis was performed across five frequency bands.	Vessel density, perfused vessel density, and percentage of perfused vessels were significantly lower in the lesions compared with contralateral healthy skin. In contrast, the contributions of different FM frequency components did not differ significantly between lesion and control skin.
[80]	10 men with lepromatous leprosy vs. 10 age- and sex-matched controls	Wrist dorsum	LDF was measured for 20 minutes, and FM was assessed using spectral analysis across five frequency bands.	No significant differences in FM amplitude were observed across the endothelial, neurogenic, myogenic, respiratory, or cardiac components between groups.

Discussion

This review analyzed findings from 41 relevant studies that assessed FM across a range of disease states using LDF. Across conditions, FM patterns demonstrated disease-specific alterations, most commonly within the endothelial, neurogenic, and myogenic spectral bands. These findings highlight FM as a potentially useful marker of microvascular regulatory dysfunction and as a non-invasive tool for characterizing microvascular health. 

Several recurring physiological themes were identified across the included literature, with one of the most prominent patterns being impairment of endothelial-related oscillations (approximately 0.005-0.021 Hz). These reductions were widely reported across metabolic and endocrine disorders, including studies in T1DM, T2DM, and CKD, where endothelial-associated oscillations were either reduced at baseline or demonstrated significant blunted increases following physiological challenges such as post-occlusion hyperemia or exercise [45,47,48,52,63]. Similar reductions were reported in hypercholesterolemia following Ach iontophoresis [42], in SS, where responses to Ach and SNP were markedly diminished [72], and in asthma, where endothelial oscillatory components were significantly reduced at rest and during hyperemia [77]. 

In addition to endothelial dysregulation, alterations in neurogenic oscillations were observed, particularly in conditions that impair the autonomic nervous system. For instance, neurogenic oscillations were consistently reduced in diabetes with neuropathic involvement, with several studies demonstrating significantly lower power in the neurogenic frequency band compared to healthy controls [56,58,59]. Similar findings were observed in patients with SCI, in which both neurogenic and myogenic amplitudes were markedly decreased during vasodilation, particularly in those with complete injuries or lower physical activity levels [65-67]. Neurogenic abnormalities were also reported in schizophrenia, where patients demonstrated abnormal PORH responses and impaired recovery of low-frequency oscillations [78,79]. 

Alterations in myogenic oscillations were also identified across several included studies. Myogenic spectral power was significantly reduced in CKD, with both baseline and post-ischemic myogenic activity lower than in controls, despite preserved peak perfusion and total hyperemic flow [63]. Similar reductions were observed in individuals with SCI, especially in those with complete injuries or lower physical activity levels, in which decreased myogenic amplitude was noted, accompanied by impaired neurogenic regulation during vasodilation [65-67]. In contrast, findings in diabetes were mixed, with some studies demonstrating preserved myogenic oscillations despite endothelial and neurogenic impairment [58] and others reporting no significant differences between early-stage diabetes and healthy controls [57]. These differences may reflect disease severity or compensatory mechanisms that maintain smooth muscle activity in early dysfunction. Beyond these abnormalities, several studies also noted compensatory shifts in higher-frequency respiratory and cardiac oscillations, particularly in advanced vascular disease and SCI, although the significance of these changes remains unclear. 

Collectively, these findings highlight the potential clinical relevance of FM analysis as a sensitive marker of microvascular health. In many of the included studies, abnormalities in FM were detectable even when traditional perfusion measures appeared normal, suggesting that FM may capture early functional impairment before structural microvascular injury is evident. This has several implications for disease monitoring, as changes in spectral components reflect alterations in regulatory pathways that may evolve over the course of disease progression. Additionally, improvements in FM following interventions, such as revascularization or therapeutic modulation, support its role as an outcome measure to track treatment response and vascular recovery. Together, these findings demonstrate that spectral FM analysis can serve as a promising tool for early detection, risk stratification, and assessment in microvascular disease. 

Aside from these findings, several limitations across the included literature were noted. The studies demonstrated substantial variability in measurement protocols, including differences in recording duration, probe placement, and environmental temperature control, which can contribute to inconsistent findings when making direct comparisons. Sample sizes were often small and predominantly cross-sectional, limiting the ability to establish causality or assess the predictive value of FM metrics over time. Additionally, definitions of spectral frequency bands varied across studies, and not all studies reported standardized stimulation protocols, such as post-occlusion hyperemia or skin-heating challenges, thereby reducing methodological uniformity. Future research should focus on standardizing the measurement of FM and on determining whether these spectral changes accurately predict clinical outcomes or treatment responses. Larger studies are important for confirming reproducibility and understanding how FM could be used in clinical practice.

## Conclusions

A review of 41 studies that evaluated FM across a range of disease states identified a variety of fairly consistent alterations in endothelial, neurogenic, and myogenic oscillations, which may be related to various aspects of microvascular regulatory mechanisms. These patterns suggest that FM analysis may provide insight into early microvascular dysfunction that is not captured by traditional perfusion measures. Spectral FM may represent a valuable approach for assessing microvascular health, and further refinement of study design and implementation will be important to clarify its clinical utility. Continued research is necessary to determine how FM can be incorporated into clinical practice for risk stratification, disease monitoring, and treatment assessment.
